# Children and parents’ perspectives of the impact of the COVID-19 pandemic on Ontario children’s physical activity, play, and sport behaviours

**DOI:** 10.1186/s12889-021-12344-w

**Published:** 2021-12-13

**Authors:** Monika Szpunar, Leigh M. Vanderloo, Brianne A. Bruijns, Stephanie Truelove, Shauna M. Burke, Jason Gilliland, Jennifer D. Irwin, Patricia Tucker

**Affiliations:** 1 Health and Rehabilitation Sciences Program, Faculty of Health Sciences, University of Western Ontario, London, Ontario Canada; 2 School of Occupational Therapy, Faculty of Health Sciences, University of Western Ontario, London, Ontario Canada; 3 School of Health Studies, Faculty of Health Sciences, University of Western Ontario, London, Ontario Canada; 4 Children’s Health Research Institute, Lawson Health Research Institute, London, Ontario Canada; 5 Department of Geography, Faculty of Social Sciences, University of Western Ontario, London, Ontario Canada

**Keywords:** Physical activity, COVID-19, Children, Physical distancing

## Abstract

**Background:**

The COVID-19 pandemic and associated public health measures have resulted in the closure of many physical activity-supporting facilities. This study examined Ontario parents’ and children’s perspectives of COVID-19’s impact on children’s physical activity behaviours, return to play/sport during COVID-19, as well as barriers/facilitators to getting active amid extended closures of physical activity venues.

**Methods:**

Parents/guardians of children aged 12 years and under living in Ontario, Canada were invited to participate in an interview. 12 parent/guardian and 9 child interviews were conducted via Zoom between December 2020 – January 2021, were audio-recorded, and transcribed verbatim. Thematic content analysis was undertaken to identify pronounced themes.

**Results:**

Themes for both parent and child interviews fell into one of three categories: 1) barriers and facilitators for getting children active amid COVID-19 closures of physical activity-supporting facilities; 2) changes in children’s activity levels; and, 3) perspectives on return to play/sport during and post-pandemic. Various subthemes were identified and varied between parents and children. The most common facilitator for dealing with children’s inactivity voiced by parents/guardians was getting active outdoors. Parents/guardians noted their willingness to have their children return to play/sport in the community once deemed safe by public health guidelines, and children’s willingness to return stemmed primarily from missing their friends and other important authority figures (e.g., coaches) and sporting events (e.g., tournaments).

**Conclusions:**

Findings from this study could inform families of feasible and realistic strategies for increasing children’s physical activity during community closures, while also providing public health experts with information regarding what supports, or infrastructure might be needed during future lockdown periods and/or pandemics.

**Supplementary Information:**

The online version contains supplementary material available at 10.1186/s12889-021-12344-w.

## Background

During childhood (0–12 years), children often explore various forms of movement via activities such as sports, playing outdoors, and engaging in play with others [1] These activities are very important for children in their developing years, as engagement in sport and/or unstructured play often leads to higher levels of moderate-to-vigorous-intensity physical activity (MVPA), which is associated with numerous health benefits [2, 3]. In fact, the Canadian 24-Hour Movement Guidelines advocate that children aged 3–12 years should achieve at least 60-min per day of MVPA per day [4] as this supports various aspects of health, including psychosocial and mental health outcomes [2], the development of motor and fundamental movement skills [4], and stronger immune systems [5]. Collectively, there is growing research to suggest that children who meet guidelines possess better overall health compared to those who do not [4].

On March 11, 2020, the World Health Organization (WHO) declared COVID-19 a global pandemic [6]. Due to COVID-19’s high transmission rates, many countries, including Canada, enforced multiple public health measures such as physical distancing (e.g., maintaining a distance of 2 m from others), mandatory masking, and lockdowns periods (e.g., requiring citizens to stay home) to reduce the spread of the virus [7] . Specifically in Ontario, these rules included many additional prohibitions, including, but not limited to: bans on indoor and outdoor gatherings; closures of facilities where people regularly gather (e.g., community centres, gyms, parks, trails, shopping malls); and, closures of schools and other large institutions (i.e., workplaces and non-essential businesses [8];). These new rules, regulations, and policies led to the restructuring and/or termination of many activities that previously supported physical activity for individuals of all ages, including children [9]. For example, organized sports were cancelled for many months (i.e., March – September 2020; November 2020 – April 2021 [8];) in staggered formats (i.e., re-opened in certain jurisdictions across Ontario as case counts dropped) due to their increased likelihood of having multiple individuals in close proximity, and outdoor play spaces (e.g., parks, splash pads) were closed to prevent large gatherings [9]. As a result, from March 2020 – January 2021 (data collection for the larger “Return to Play” study), many children and their families no longer had access to typical physical activity-supporting environments and programming, thus resulting in many barriers to ensuring children were sufficiently active each day. Moreover, over the last 18 months, the government of Ontario has adopted various re-opening strategies; however, at the time of data collection, a colour coding system was in place [8]. For example, Ontario jurisdictions were categorized as being either in the grey, red, orange, yellow or green-zones, dependent on the number of active COVID-19 cases in the area [8]. Colours were used to inform citizens about the activities that were allowed and/or prohibited, and to keep jurisdictions aware of the risk in their respective communities.

Prior to the COVID-19 pandemic, the proportion of Canadian children meeting national integrative movement behaviour guidelines (i.e., physical activity, sedentary behaviour, and sleep) was already problematic. In fact, only ~13% of Canadian children aged 3–4 years and 5–12 years were meeting their respective age-specific guidelines [10, 11]. Unfortunately, these low rates have been further compromised by the pandemic – recent data from early pandemic days (i.e., April 2020) showed that only 2.8% of Canadian children were meeting the 24-Hour Movement Guidelines [12]. Moreover, preliminary findings have demonstrated the negative impact of COVID-related lockdowns and closures of outdoor spaces on Canadian children’s physical activity opportunities [12] and levels [13]. For example, Moore and colleagues’ study found a dramatic decline in children’s outdoor physical activity, and drastic increase in screen time and social media use [12]. Similarly, Bates and colleagues’ [13] commentary also reported significant decreases in physical activity, increases in sedentary time, and sleep disruptions among children aged 5–17 years. These trends are apparent worldwide, as studies in Italy [14], South Korea [15], Shanghai [16], and the United States [17] have reported similar behavioural shifts among children during COVID-19. Undoubtedly, lockdowns and closures of physical activity-supportive spaces, despite their positive role in helping to curb the spread of the virus, have also had negative impacts on children’s movement behaviours and have posed difficulties for parents and guardians who are trying to support their children’s health.

To date, limited qualitative studies are available examining how the COVID-19 pandemic has impacted families and children as it relates to physical activity [18]. Further missing from the literature are children’s voices. More specifically, a lack of information exists regarding children’s perspectives about getting active during COVID-19, their experienced barriers, as well as the facilitators implemented by this young cohort. Thus, the primary purpose of this study was to explore Ontario parents’ and children’s perspectives of the COVID-19 pandemic’s impact on children’s physical activity behaviours, perspectives on return to play/sport, as well as the barriers and facilitators to getting active amid extended closures of physical activity venues.

## Methods

### Study design

This study was part of a larger cross-sectional study, entitled ‘Return to Play’, which quantitatively assessed the perspectives of parents during the pandemic (i.e., August – December 2020) regarding their children’s physical activity levels, their intentions to resume sports and physical activities post-pandemic and their risk tolerance. To solicit richer, contextual data, the present study captured qualitative data via interviews about physical activity participation during the pandemic from a pool of survey participants who expressed interest in participating in online Zoom interviews. The Western University Non-Medical Research Ethics Board approved all study procedures and associated documents (REB #116331).

### Recruitment and participants

Parent participants were eligible to participate if they were a parent or guardian of a child 12 years old or younger, resided in Ontario, had custody of their child(ren) at least 50% of the time, and participated in the larger study (i.e., online survey). Upon completion of the survey, participants were asked if they were interested in participating in an internet-based interview (i.e., audio only, conducted via Zoom) to further share their perspectives on their children’s return to physical activity-related programming post-COVID-19, and activity levels during COVID. A total of 1097 parents completed the online survey and were included in the original study, and 392 parents expressed their interest in participating in a follow-up interview. Using www.randomizer.org, parents were randomly selected from the 392 volunteers and were invited via email to participate in an interview. At this time, parents were also notified that their children were invited to participate, if interested. Only children that met the inclusion criteria (i.e., 12 years of age or under, living in Ontario, had a parent who completed the online survey, and spoke English) were eligible. If participants did not respond to the email within one week, the next randomly selected participant was contacted and invited. A total of 32 parents and their children were invited to participate. Recruitment continued until saturation was achieved.

### Interview procedures

Data collection occurred between December 2020 and January 2021. All parent interviews were 30–45 min in length and were recorded via the online video platform, Zoom. Child interviews were completed subsequently to parent interviews if from the same household, or uniquely if not (i.e., parent who completed the online survey organized a time via email for their child’s interview to take place with the research assistant). Dependent on the age of the child, a parent was sometimes present for the duration of the interview. All child interviews ranged between 10–25 min in length. Only audio content for both parent and child interviews were recorded. Individual interview guides were created by the research team for parent and child participants. Parents/guardians were asked to provide verbal consent at the start of interviews, and verbal assent from children was also obtained. Member checking (i.e., restating or summarizing information for the purpose of determining correctness) was used by the research assistant conducting interviews to ensure accuracy of participant responses [19], verify the trustworthiness of qualitative results [20] and ensure credibility [21]. Interviews were digitally transcribed verbatim using QSR NVivo (version 12). Two trained research assistants confirmed the transcripts for accuracy using NVivo. Upon completion of each interview, participants were given token of appreciation for their time. Please see Additional file 1: Appendix A and B for the parent and child semi-structured interview guides, respectively.

### Analysis

All interviews were analyzed using QSR NVivo (version 12). Thematic content analysis was conducted by two trained researchers to ensure confirmability and to identify common participant responses [22, 23]; specifically, the records of all transcripts coding processes were documented, to ensure a logical and traceable approach [21]. In addition, intercoder reliability was assessed using reliability checks during the analysis period (e.g., reviewing evidence and debriefing [22];), and comparing codes in Nvivo. Because the interviews were conducted using semi-structured interview guides, transcripts from parent and child interviews were analyzed separately and responses were grouped based on each question and analyzed deductively. Thematic analyses were conducted in accordance with Braun and Clarke’s best practices [23]: (1) read through all interview transcripts to become familiar with the data, reviewing the content repeatedly; (2) generate a list of codes based on items found in the data sets, and colour-coded items manually when patterns emerged; (3) analyze codes and identified prominent themes and subthemes; (4) review the themes; (5) identify proper names for themes; and, (6) present interview data via tables and written summaries of the themes and subthemes using quotes. Deductive content analysis was used to test existing categories, and concepts that became apparent to the research assistants from responses to open-ended questions in the online survey. Confirmation bias was avoided as the secondary coder was only responsible for coding interviews and was not directly involved in the project [24]. Having two independent coders prevented the occurrence of having the research team form a hypothesis or belief and using the respondents’ information to confirm that belief.

## Results

A total of 12 parents/guardians and 9 children from across Ontario participated in interviews. Nearly all (*n* = 11, 92%) of parent participants identified as female and were from urban communities. Parents had an average age of 40.67 years (*SD* = 7.49), and an average of 1.67 children (*SD* = 0.651). Majority (n = 8; 67%) of parents lived in semi-detached or detached homes. Child participants were mostly female (*n* = 6, 67%), had an average age of 7.33 years (*SD* = 2.92), and all resided in urban environments across Ontario. See Table 1 for full participant characteristics.

**Table 1:** Participant Characteristics of Parents/Guardians (*n* = 12) and Children (*n* = 9) in Ontario

Participant Characteristics	Parents/Guardians		Children	
	***M***	***SD***	***M***	***SD***
Age (years)	40.67	7.50	7.33	2.91
	***N***	**%**	***N***	**%**
Gender				
Male	1	8.3	3	33.3
Female	11	91.7	6	66.7
Community Type				
Urban	11	91.7	9	100.0
Rural	1	8.3	–	–
Colour/Phase Re-Opening (at time of interview)				
Yellow	1	8.3	–	–
Orange	–	–	1	11.1
Red	5	41.7	3	33.3
Grey (Lockdown)	6	50.0	5	55.6
Housing Type				
Apartment/Condominium	2	16.7	1	11.1
Detached or semi-detached house	8	66.6	7	77.8
N/A	2	16.7	1	11.1
Number of Children				
1	5	41.7	–	–
2	6	50.0	–	–
3	1	8.3	–	–

Note. Colour/Phase Re-Opening refers to the current state of Ontario’s re-opening plan at the time of data collection (i.e., December 2020 – January 2021). Please see https://www.toronto.com/news-story/10239506-here-s-how-ontario-s-covid-19-colour-codes-work/ for more information

Analyses revealed four over-arching themes which were apparent in both parent and child interviews, and were consistent with the questions from the semi-structured interview guides: (1) barriers associated with children getting active during COVID-19-related closures; (2) facilitators used to encourage children to become active during COVID-19 closures; (3) perspectives on returning to organized sport and/or engaging in physical activity involving social interaction with others during (e.g., when permitted in Ontario; physical distancing and wearing personal protective equipment); and, (4) changes in children’s physical activity levels during COVID-19. Unique to parent interviews, two additional themes were social connectedness and lack of regular access to sport and supportive environments (e.g., parks, playgrounds). See Tables 2 and 3 for sample quotes for each theme and subtheme from parents/guardians and children, respectively.

**Table 2:** Parents’ Experiences (i.e., Barriers, Solutions) of Getting their Children Active During the Pandemic and Perspectives on Return to Play/Sport

Question	Theme	Example Quotes
What **challenges** did you experience with getting your children active while at home?	Closures of Supportive Spaces	- “At one point in [city] where I am, they actually closed hiking trails. They came and they put big like boulder things like big cement blocks. That was probably the most upsetting thing because I was like… this is the last thing that we can do outside.” - “He would just look at you and he’d say, can’t go there [outside], Mom, because the [in]fection, that’s what he calls it, I know, and the [in]fection. Yeah. And it was heartbreaking.” - “We would go for walks by the park and just had to try to explain to her that they had closed it, but it was a little bit heartbreaking for us because we knew that as she got older, she was more able to play and use these to use the equipment. So, it was hard to not allow her to do that…”
	Weather	- “Now that it’s getting a bit colder and now that we returned to work, I don’t feel like she’s getting nearly enough activity.” - “In the summer, we’re very, very active. But yeah, in the winter now, like now it’s getting hard again because I will get up and get bundled up and take them out. But my four-year-old wants to wear shorts all day, every day. I can’t, like he fights every morning when I try to take him to school to put splash pants or snow pants on, even like he hates it. So, he just refuses to get ready to go outside.” - “It was hard to get them outside to play where they wouldn’t freeze after 5 min.” - “When the rainy gray season starts, they don’t really want to go outside.”
	Lack of Motivation	- “It’s a lot of onus on me, like I said, in front of the computer all day for work, I’ve got a really high, high stress job that is very difficult. And the last thing I want to do is like I don’t want to have to be the instructor or the teacher on the computer again.” - “So, am I giving her as much experience as what she would be getting at school or sport? Definitely not, you know. I don’t have the energy to do that.”
	Financial Implications	- “I looked into, you can swim, which is allowed if it’s a private lesson, but it’s one on one, but it was prohibitively expensive. So, I wanted to do it, but I can’t afford it.” - “We tried to purchase some aids in the form of like a climber or like a swing set or bike but like literally everything was sold out like or anything that was left available was like exorbitantly priced.” - “I also become a little bit resentful about spending the money on that. I’m all I want to support small businesses, but I, I have two kids enrolled and I’m paying two hundred dollars a month for them to rent somebody dancing [with regard to online classes].”
	Disruption and Change to Routines	- “Oh, it was really hard. We fell into a pretty unstructured routine, I guess. Bedtimes were later. Sleep ins were later.” - “I got really, really lax with the routine. So, they were doing whatever they wanted to do more often. And I just I had to kind of let go of the control and let go of what I thought a successful day was going to look like.”
	Increase in Screen Time	- “My kid plays Fortnite and he does a lot of gaming and he’s on the computer a lot. So, I really struggle with trying to get him off of the technology because it’s been so much more accessible to him. So, trying to get him outside, even to play basketball, to go sit in the backyard, to do something outside, it’s a lot harder now.” - “We’re going through a lot of temper tantrums, fits, him being very angry because I’m trying to take the devices away…” - “Screen time has increased totally. Because at the beginning, there was nothing for you really to do but to watch TV. There was nowhere to go. Nowhere to go and really nothing to do sort of thing.”
	Quality of Virtual Instructions	- “She found it really discouraging. In fact, how they had done it was pre-recorded. It wasn’t like a Zoom live type dance session. So, she got really frustrated that the instructor couldn’t see her or wasn’t speaking back to her or that like, you know, she’s not making suggestions like, can we do this? Can we do that? And it was so regimented.”
	Housing Type (i.e., Presence or Not of Outdoor Space)	- “We have no yard; we have no balcony. And our neighbor in the summer at one point just said, look, if you want to use our backyard, you can come sit here, which made a huge difference.” - We found it very challenging. So, we live in a townhouse that does not have a basement or backyard. We had to try to utilize just the front yard space, which isn’t impossible. But it just made it difficult because we’re right on a street, so then we were scared because we are on a corner lot that is adjacent to the exit of a cul, kind of like a cul de sac sort of thing. So, people come out of there with the vehicle.”
What **solutions** did you undertake to deal with these challenges?	Engaging in Activity Indoors	- “We set up obstacle courses with like furniture in our house like, I don’t know, unsafe probably things. But we had fun and yeah. And we did dances and songs…” - “Like we have like a Google Play, and I would let them use Spotify and they would just dance around inside to random kid songs…”
	Bubbling with Neighbours and Other Families	- “We have a communal courtyard outside our house. And I think once the sun came back, there was one family that sort of approached my son and they were like, are you OK if they play together?... And sometimes some of the kids wear masks, some don’t.” - “I’ve actually networked on like via Instagram and Facebook and things like that. I’ve actually been able to network with quite a bit of other moms that are that feel the same way I do about everything and want their kids to be active and want their kids to socialize. So, we have like playdates and stuff at the parks and like we’ll meet up outdoors and try and let the kids run around and still socialize and things like that. So that’s been a godsend.”
	Getting Outdoors	- “We have amazing greenery in the area. There’s lots of trails and creeks and all that kind of stuff. We’re right by the lake. So, there’s a ton of nature outdoor stuff to do in the summer. Thank goodness.” - “We went hiking every single week. Lots of activity, lots of outside time. With so much international travel and like cross provincial travel, we never really explored Ontario. We had no idea what was close by and we’ve hiked everything now close by so well, when you think you’ve done it all, there’s more to do with it. So that was a radical change. We started to explore our own backyard. You know, lots of good changes for the future.”
	Virtual platforms	- “There are all these free programs available on YouTube and stuff to get them up and dancing.” - “They would they found this YouTube channel where they could do kids exercises. So, they would do that every day just to kind of keep them off the screens and active.”
	Housing Type/ Presence of Outdoor Space at Home	- “We are so lucky that I have a big yard, so we have like the trampoline in the back. A yard to play in the front yard, all that kind of stuff, so it definitely helped having an outdoor space.” - “…like when I’m working, I’m comfortable running them on their own in the backyard.”
Do you intend to make any changes to your child(ren)‘s active play/sports programming as a result of the pandemic?	Children’s Views on Masks and Physical Distancing Rules	- “I think when people think that, like, oh, kids can’t wear masks or kids won’t be able to do this. The kids actually have no problem with it. They’re taking their cues from us. Kids know what’s normal based on what everyone else is doing around them. So, it’s really just when their parents are against it, they have a big issue with it. And pre-COVID kids had to wear masks in the hospital, sometimes for various reasons. And we never had a kid freak out. They just did it, all the nurses did it. So, they did it.”
	Willingness to Have Children Return to Play	- “I think that risk is so low that it’s, it’s an acceptable level of risk because the risk to my child not being able to access physical activity and all the developmental goodness that comes with interacting with his peers in this kind of group setting, that’s a greater risk to his health than the very small chance that he contracted COVID or something else.” - “I would like to get back to a situation where kids are allowed to play with each other and touch each other and be close as quickly as possible, and as soon as anything like that is offered, I will be signing my kids up.” - “I’m more worried about how it’s being dealt with and what it does for kids to be told to stay away from each other, to wear masks when they see other people. Like I find that the potential psychological impact on kids to be more dangerous.”
	Physical Distancing Rules	- “One of the neighbor’s child is in hockey, and he doesn’t want to go because of all the protocols, because it’s not as fun.” - “I’m very comfortable with what they’re doing at hockey. I mean, everyone only one parent is allowed in the arena with the kid. There’s only ten people allowed the dressing room. Everyone has to wear masks…”
What are your **overall feelings** regarding your child(ren)‘s eventual return to play/sports?	Lack of Motivation to Parent	- “I just really I had to just focus on me, like, really, I had to, like, maintain my mental health a little bit. I just I had to kind of let go of the control and let go of what I thought a successful day was going to look like.”
	Parents’ Reduction in Activity	- “And for both my husband and I, were both super into running... but since Sunday, neither of us have even ran one kilometer, you know what I’m saying? So, like, it just it shows you how much the pendulum swings for sure. It was good up until we were really isolated.”
	Children’s Social Connectedness	- “So, you know, I think [child] has some mental health problems, because now with all this happening, you know, everyone’s getting on each other’s nerves, and I know [child] getting frustrated with us and we’re getting frustrated with each other because we are trying to be careful and trying to go by most of these guidelines.” - “They went through this stage where they were like, does it really matter? You know, nothing’s going to go back to normal like, you know, week after week, you know, just kept continuing and just knowing that school could not come back so they really just the school not going back to, like, what’s going on so they were like, who cares? And I just I took it easy on them. You know, because they are children still and, you know, everybody was going through the same thing. But this is really new for a child that was go, go, go. And all of a sudden, you’re, like, you know what? You don’t have to go anywhere. You stay at home and watch the iPad if you want to, or you could watch TV if you want is it was an adjustment for them.” - “There would be like an episode where she might get upset and she cries, and that hurts your feelings, too, because she’s justified and you don’t know how else to explain it to her, and she just has to get through it and but you’re like, there’s no reason for this (…) She’s not getting in trouble, but you’re kind of giving her so many rules and all she just wants to do is just throw the ball and just sort of be free, so. It just maybe stifles her a little bit and then can cause a little bit of frustration, (…) we had to deal with a lot of that.”
	Parents’ Social Connectedness	- “Well, I guess when you’re at the dance studio five nights a week and then all of a sudden you’re not there anymore.... it’s like for her, it was more like the loss of activity. For me, it was I feel it was my that was my social time.” - “I’m glad that this is my second child and not the first one because we were so lucky to be able to be a part of so many, like mommy and baby groups before. I think it was really beneficial for her and now we can’t do that with our second.”

**Table 3:** Children’s Experiences (i.e., Barriers, Solutions) of Getting Active During the Pandemic and Perspectives on Return to Sport

Question	Theme	Example Quotes
What **challenges** did you experience with getting active while at home?	Not Being Able to See Important Personnel (i.e., Friends, Coaches)	- “It impacted me like, very much, because I couldn’t see my dance friends. I couldn’t go to dance, and I couldn’t do much.” - “I was trying to get used to not going outside, not going to the park, and not seeing my friends but it was really hard.” - “I miss seeing my friends and getting to go to competitions or swim meets and just having fun.” - “I miss seeing how people can teach me new ways to get better at stuff or like to play with a group of people instead of just one person.”
	Engaging in a lot of Screen Time	- “I’d pretty much be sitting on my phone talking to my friends all day.” - “We watched a lot of tv shows, YouTube, and TikTok.”
	Lack of Things to do at Home	- “It’s challenging not being able to play with my family because they’re like doing work and stuff and because my dad still goes to his job.”
	Closures of Outdoor Spaces and Sport Facilities	- “I can’t go on the slide, and they took off? the swing, the monkey bars and the rings.” - “When you get to be in person is much more fun than trying to dance on your own at home when you don’t have the studio, or mirrors, or space.”
What **helped you to** deal with these challenges? [solutions]	Maintaining Sport or Activity Engagement at Home	- “I had some online gym classes, and I would go outside and roll out my mat. I’d go on my trampoline, and my friend made me download this workout app so I’d workout with her on the phone.” - “I played hide and seek with my sister. We also played tag, skipping rope, hula hoop and hopscotch.” - “I miss basketball, but me and my mom play basketball together.” - “I have a hockey set-up in my basement, so I play hockey in the basement.”
	Being Able to See Others Outside	- “When things started to cool down, I would be allowed to go hang out with a few friends that we like, knew parents of.” - “We went and played at parks and at my school. We got outside and built an igloo.” - “I got to see the neighbourhood kids in the summer, and I made some new friends.”
	Talking to Friends Virtually and on Virtual Platforms	- “We made a group chat, and we’ll play games together and stuff and talk about what we did, what we ate, what we learned online, at school, and asking each other about what all were doing at home.” - “Knowing we were all kind of going through the same thing was helpful. And knowing that it wasn’t just me who was missing all of the activities and sports events we had lined up.” - “I used to do dancing online, but it [the internet] was always freezing.” - “I went on hockey training camp on Zoom five days a week, 1 h per day, for 6 weeks.”
Have you and/or your parents/guardians already **made any changes as a result of COVID-19** to your active play or sport programming? For example, are you back in any team activities or planning to go back?	Parents as Primary Decision Makers	- “My mom’s thought process was like if were going back to school, and they want me to keep my social bubble, I’m already exposed to the same people. Dance isn’t going to be much worse, because it’s going to be the same precautions as school. And a lot of my dance friends are also from school.” - “I’m not the one who decides... it’s my mom.” - “After corona, I’m planning to go back only if my mom lets me, and now that its corona, no, I’m not planning on doing anything.” - “We heard that dance was coming back, and we just went back. It was like a no brainer.”
	Perspectives on Health Protocols at Sport	- “It was still super easy with the protocols.” - “I don’t mind wearing my mask.” - “It’s kind of the same as before besides the 5 or 10-min period for them to clean. So instead of having 45-min classes, we had 35-min classes, but we still got the same amount of dance in, we just had to go a bit faster.” - “It was harder because I kept losing my mask. I couldn’t keep track of it at gymnastics.” - “At hockey, there’s a usually a guy waiting at the door and he’s going to ask you questions about if you were out of the city and if you have any symptoms of Covid and then you would go in.”
Compared to your routine before the pandemic started (as a reminder, this was in March), do you think you were more or less active back then than you are now?	Reduction in Activity Levels	- “I was more active before because with Covid you can’t do very many activities.” - “I miss that I used to be really energetic in dance and I’m sad I missed my dance recital.” - “I thought I was just going to, like, not be active enough to go back [to dance]. But I think other girls felt the same too.” - “I’m less active because we don’t have any games. We’re just doing drills.”

Salient differences in themes were observed across parent and child interviews (i.e., unique subthemes emerged). For example, with regard to barriers to engaging children in physical activity, parent interviews revealed weather, housing type (e.g., apartment, detached house), screen-time, loss of their previously daily routines, financial barriers, closures of supportive environments, and lack of motivation as subthemes, while children perceived missing important people (i.e., friends, coaches), closure of outdoor play spaces (i.e., parks) and lack of things to do at home as the most notable barriers. In greater detail, many parents reported that screen time became an overwhelmingly large part of children’s days, as they had “nothing else to do” while confined at home for extended periods. In addition, finances were mentioned by parents as a barrier towards promoting their children’s movement during the deeper stages of the pandemic (i.e., fall of 2020), when the novelty of spending copious amounts of time at home began to ware off. Parents noted this barrier arose as Ontario began to re-open following the first wave (i.e., March – July; 2020 [8];), and sport facilities were permitted to operate at lower capacity. Finances were mentioned with regard to both the purchasing of physical activity-promoting equipment (e.g., trampolines, bikes), as well as the increased cost of sports (when deemed accessible) due to a switch to private lessons, and costs of virtual classes. Finally, the loss of daily routines and lack of parental motivation to support children’s activity was frequently noted and was exacerbated by periods of cold weather which posed additional challenges.

With regard to facilitators to getting children active during the pandemic, sample subthemes from parent interviews included: bubbling (i.e., clustering with people outside of immediate household) with other families, spending time outdoors, and using virtual platforms (i.e., Youtube and Tik Tok), of which the latter two were also noted by children. Parents spoke more about the influence of their living space and community (i.e., rural, urban) on their ability to facilitate their children’s activity, whereas this was not frequently mentioned by children. Regarding common outdoor spaces, parents with free standing homes that had backyards and other outdoor spaces (i.e., communal living areas, courtyards outside apartments) reported these spaces to be instrumental in their ability to promote their children’s movement. In addition, children who had outdoor play spaces and equipment (i.e., trampolines, yards) that facilitate activity, referenced using them often to maintain their sport skills. Finally, children alluded to getting active with their parents whenever possible, and those who had siblings expressed their engagement of play with them as a facilitator for getting active during stay-at-home orders.

During child interviews, children were most inclined to engage in conversation about what they missed concerning their pre-COVID activities. The most frequently uttered phrase was “I miss my friends”. Furthermore, similar to missing friends, children also reported that they missed other important people in their lives, such as their coaches and mentors, and events that come alongside sports (e.g., tournaments). Children reported that they missed their coaches because they felt like their skillset for their sport (i.e., hockey, dance) was falling behind without having constant support and training from someone deemed an “expert”. As a result, it was not surprising that nearly all children who had returned to sport at some point prior to interviews, reported having no issues with following health protocols. Children reported that wearing their mask was not a burden, and that returning to sport/play was their priority. Children also commented on enhanced cleaning procedures at their respective sports, not being able to change in changerooms (i.e., coming prepared to sport), and hand sanitizing. Overall, no major concerns were noted by children about health protocols. Similarly, parents reported being satisfied with public health guidelines and health protocols at sports, once they had re-opened, in their respective Ontario cities.

Finally, parents emphasized their loss of social interaction at children’s event (i.e., dance lessons, tournaments) as a social connectedness consequence of sport closures, for both themselves and their children. Parents reported that having their children at home for extended periods of time without any social interaction worried them about the impact of the pandemic on their children’s social development. In addition, parents reported missing their own time to socialize with other parents at their children’s sporting events. Finally, with regard to changes in physical activity levels, both parents and children reported that children’s levels of physical activity declined during the pandemic. Specifically, it was noted by parents that they felt their children’s engagement in MVPA declined, and that they were no longer getting nearly as much heart-pumping or sweat-inducing activity. However, younger children reported being less influenced in regard to their activity levels by COVID-related closures and loss of programming.

## Discussion

The purpose of this study was to explore the perspectives of Ontario parents/guardians and children regarding their experiences getting children physically active during the COVID-19 pandemic, and to examine their feelings concerning children’s return to physical activity-related programming. The impact of the pandemic on physical activity levels of children was also explored. Given the timing of the interviews (December 2020 and January 2021), some parents reported that their children had already returned to sport (during a reopening in summer/fall 2020) and were required to follow health protocols (i.e., mask wearing, not being able to access change rooms), while others had not yet returned, as this was dependent on where they were located in the province (i.e., colour phases of re-opening happened at the local level, not provincial [8];). Overall, parents and children provided a great level of detail regarding their experiences during the COVID-19 pandemic. Many themes were identified, and centered around common barriers (e.g., closures of supportive environments), facilitators (e.g., virtual platforms) and perspectives on return to play/sport, including parents’ and children’s views of new health protocols, lack of motivation to get active during periods of lockdown, and personal protective equipment. It was clear that the pandemic greatly influenced children’s movement opportunities and behaviours, as noted by parents and children. Several findings are discussed below.

When asked about challenges regarding children’s physical activity participation amid COVID-19 closures, parents frequently referenced the initial stages of the pandemic (i.e., March to May 2020). Parents expressed that these were the most challenging months to encourage behaviours that supported their children’s movement, because of the uncertainty associated with engaging in activity outdoors (e.g., potential risk of aerosol transmission at the park), as well as the cold weather in Ontario. Certainly, being confined to one’s home and trying to support children’s activity while indoors was a commonly noted challenge by parents. In addition, parents who did not have backyards or outdoor space tucked away from major roads voiced their frustration, as they previously relied on parks and/or neighbourhood spaces to safely support their children’s movement pre-COVID-19. This is consistent with research conducted prior to the COVID-19 pandemic that supports the impact of the neighbourhood environment on the extent of children’s physical activity [25], as well as research comparing urban and rural living environment’s effects on physical activity levels of Croatian adolescents during COVID-19 [26]. As most participants in the present study were from urban environments, references to outdoor and indoor spaces and their influence on physical activity offerings were frequently noted. Recently, Zenic and colleagues [26] found a significant influence linking adolescents living in urban environments with lower physical activity levels during the pandemic, compared to their rural counterparts. It is interesting to note that Zenic et al.’s [26] findings report that adolescents living in urban settings had physical activity levels that were lower due to their dependence on organized sport for movement, consistent with our findings.

Regarding common outdoor spaces, parents and children with outdoor spaces (e.g., backyards, communal courtyards) reported these spaces to be instrumental in their ability to support movement. Specifically, children who had outdoor play spaces and equipment (i.e., trampolines) that facilitate activity, referenced using them often. This underscores the importance of the home environment, and presence and/or lack of outdoor space during a global pandemic. Consistent with Mitra et al.’s findings, parents with children aged 5–11 years who lived in an apartment during the pandemic reported that their home space discouraged healthy movement behaviours [27]. In addition, living in neighbourhoods with low density and further from major roads (i.e., highways, large intersections) promoted increased outdoor activity among children [27], and aligns with findings by Nigg and colleagues [28] who found children living in lower density areas exhibited more physical activity during COVID-19. Although the present study only had one participant from a rural environment, this participant noted that their family was not as affected by the closures of supportive environments, as they had their own outdoor playground due to their living space. As a result, according to this parent, living in a rural environment during COVID-19 increased the feasibility to abide by physical distancing, and reduced barriers to accessing outdoor physical activity.

Finances were noted by parents as a barrier for engaging children in activity. Specifically, parents reported sport enrollment during COVID-19 came at a higher cost, likely due to increased health measures in Ontario during phases of re-opening, where less children were permitted at a time in various sport facilities (e.g., smaller team sizes), or private lessons becoming the preference, as they were more conducive to physical distancing. In this case, enrollment costs were increased, and parents expressed their frustrations. This represents a possible barrier for future consideration to enrolling children in sport post-COVID (i.e., if costs remain increased as a result of smaller groups due to physical distancing regulations). In addition, the financial aspect of returning to physical activity-related activities post-COVID has also been identified in Australia (Elliott et al. [29],), and has made many parents reference their preference for spending time outdoors or in free, open spaces. Finally, parents emphasized that purchasing home-equipment was a strategy for maintaining their children’s activity, but also came with financial implications and budgeting for new toys.

Screen time was another commonly noted barrier to engaging children in activity. This is consistent with Moore and colleagues’ findings that showed a drastic increase in social media use during COVID-19 [12] and Riazi et al’s study, in which parents reported a drastic increase in their children’s screen time, some referencing their children’s engagement tripled during various timepoints throughout the pandemic [18]. Interestingly, parents in the present study reported they were “just human” and that an increase in screen time was inevitable as it provided them relief from parental demands, even if just for 30 min. However, some parents also reported increased behavioural issues following removal of screens (e.g., temper tantrums), as children got accustomed to spending majority of their days without other tasks or activities. This is similar to the theme of routine disruptions noted by parents, as without regular programming (i.e., waking up at consistent times for work or school), family routines were irregular or “thrown-off” (e.g., no scheduled “school” hours due to virtual and online learning, more time watching movies, later bedtimes). Similarly, many children reported that they were aware of their increased time spent engaged in screen-viewing during periods of lockdown. Clearly, families spending copious amounts of time at home has led to many changes to daily schedules, including juggling work, parenting, and homeschooling demands, which has consequently, resulted in children engaging in increased amounts of screen-time, alongside a change in typical routines.

Having access to screen-based technology was mentioned by a handful of parent and child participants as a facilitator for activity. Some parents reported allowing their children to follow YouTube tutorials and learn online dances such as those on the social media application called TikTok. The use of virtual platforms identified in the present study is consistent with findings by Moore et al. [12] and Dunton et al. [17] who found remote and streaming services one of the most frequently used mediums by parents to engage children in physical activity during COVID-associated closures. In addition, children referenced various virtual experiences that helped them maintain activity during closures. For example, one child participant mentioned a kid’s physical activity smartphone application that provided in-depth at-home workouts and stretching, and other children referenced spending large amounts of time following and/or learning Tik Tok and/or YouTube dance routines with their family members. As a result, screen time seems to have acted as both a barrier and solution for parents and children during the COVID-19 pandemic in Ontario.

Nearly all parent participants reported that they believed their children’s activity intensities changed during COVID-19. Specifically, the majority of parent participants stated that they believed their children’s engagement in MVPA declined as a result of closures of supportive environments and organized sport, consistent with findings by Moore et al. [12] showing that children’s physical activity declined in all forms of activity (i.e., outdoor activity, sports), except for household chores. This is likely due to the time demands of engaging children in this type of activity, as many parents reported that after working long days and juggling at-home tasks (i.e., organizing children’s virtual homeschool hours) the last thing that they had energy for was engaging their children in high intensity activity. Specifically, parents noted children’s inability to play with other children as a result of stay-at-home orders, on top of inaccessibility of their regular extracurriculars (e.g., dance, hockey), as contributors to their reduced physical activity. Fortunately, parents reported that outdoor activity was extremely conducive to supporting children’s higher-intensity movement, even if all that children received was unstructured activity (e.g., running in an open field) for a short period of time. This is consistent with research that reports children are more inclined to be active when outdoors [30], and outdoor play spaces provide additional benefits [31] to supporting movement.

Consistent with their parents, children also reported that they believed their levels of physical activity declined during the pandemic. However, this was more frequently noted by children in the upper years (i.e., 7–12 years old). Children nearing early adolescence reported their fears about diminishing their skillset for their sport as a result of not having access to their usual environment (i.e., studio, arena). In addition, they noted that engaging in sport-related skill development at home was not as physically demanding as doing so in their typical sporting environment. However, younger children said they felt they were still getting into higher levels of activity during time spent at home because they could engage in sport with their parents. This finding could be a consequence of the level of sport in which the children participated, as younger children tended to be primarily involved in recreational activities rather than elite sports, or the inability of young children to accurately recall physical activity participation.

Finally, the most common facilitator for physical activity engagement that parents, and children reported engaging in during the early stages of the COVID-19 pandemic were getting active outdoors. Specifically, during the summer months, when the first wave of the pandemic was coming to an end and restrictions in Ontario slightly eased (i.e., July to August 2020), and more information regarding the safety of outdoor spaces became available (e.g., little to no risk of aerosol transmission [8];), parents reported that taking their children on hikes, playing in backyards, and exploring their neighbourhoods by going on long walks were facilitators to encouraging activity and reducing screen time. This is consistent with Dunton et al.’s [17] findings, who also reported that unstructured outdoor physical activity was the most common form of movement for this cohort during the early stages of COVID-19, and Schmidt et al.’s [32] study that reported an increase in children’s habitual physical activity (e.g., playing outside, gardening, cycling etc.) during COVID-related lockdowns. In addition, many children voiced their disappointment about being unable to play with their friends in the park during the initial months of the pandemic (i.e., when parks and outdoor recreation spaces were closed and gatherings were limited to individual households only), but reported that when playing sports outdoors and maintaining their physical engagement in community spaces (i.e., school playgrounds and fields) was deemed accessible by public health, it was a good strategy for them to engage in activity, practice their sport, and spend time with friends while abiding by Ontario’s physical distancing rules. This is important, as research shows the important role of peers in children’s physical activity [33]. Specifically, children are more likely to be active when accompanied by their friends [34], and children who report having more peers also report engaging in higher levels of activity [35]. Similarly, in the present study, children with siblings cited playing with them during Ontario’s stay-at-home orders as a means to maintain activity. These findings are consistent with Kracht & Sisson’s [36] systematic review that found children with siblings to have healthier physical activity patterns compared to only children.

As children who participated ranged from 4 to 12 years of age, it is apparent that missing friends is important for children both in their early years and those nearing adolescence. This is consistent with findings from Pelletier et al’s [37] qualitative study, that also found children aged 7–12 reporting negative feelings with regard to missing their peers during periods of lockdown. Moreover, children who had already returned to sport at the time of being interviewed specified that their parents helped them make the decision to return to sport, but it was also a collaborative approach. This is consistent with previous research that shows parents play an important role in facilitating children’s engagement in sport, as they are the individuals responsible for payment and transportation to these sorts of events and facilities [38]. Parents reported that they asked their children if they wanted to return, or if they preferred to stay at home. However, few parents reported that they felt if their child had returned to school, returning to sport was not much different with regard to contacts and/or increasing their social bubbles. As a result, both parents and children expressed their desire and comfort to return to sport if it was deemed safe by the Ontario government.

The themes identified during the interviews with parents and children show that social connectedness and interaction with friends and coaches is essential to the maintenance of children’s well-being and physical activity engagement. In addition, parents reported that having their children at home worried them about the impact of the pandemic on their children’s social development and associated risks of social isolation. This is in line with other research published regarding the implications of the COVID-19 pandemic on many children [29, 39]. For example, Elliott et al. [29] found that parents noticed their children to be more irritable and frustrated due to the lack of social connectedness with others for an extended period. Additionally, research shows that increased rates of depression and anxiety among children and adolescents have been documented during the pandemic [40]. This is important to address as physical activity has been noted to reduce mental health concerns in children [2], yet we are seeing decreased rates as a result of COVID-19 [12], which are likely exacerbating social isolation and irritability, as noted by parents in the present study. More research is needed to explore how supports can be put in place in the case of future pandemics and/or stay-at-home orders to support children’s physical activity and support their overall well-being.

### Strengths and limitations

This study represents one of the first that captured the voices of children regarding the impact of the pandemic on physical activity, play, and sport during the pandemic. However, limitations must also be addressed. First, all participants were recruited from our larger survey-based study exploring the effects of the pandemic on return to play, and so some participation bias may be apparent. Second, it is important to note that all interviews were conducted between December 2020 and January 2021, approximately 9 months after the onset of the pandemic, which may have had an influence on perspectives of return to sport (i.e., compared to if interviews were conducted at the onset of the pandemic). Third, nearly all parent participants were female from urban environments, which limits the diversity and transferability of perspectives obtained through the parent interviews. Further, no children under the age of 4 participated in interviews, thus limiting the findings of our study to this younger cohort. Additionally, the present study was only focused on residents of Ontario, therefore, perspectives may vary in other provinces/territories where COVID-related restrictions were different. Finally, because participants were recruited via randomization (due to the extremely high interest expressed post-survey), and no ability to recruit a diverse sample for interviews (as their sign up was not tied to their survey responses/data), most participants were from urban environments.

## Conclusion

The effects of COVID-19-related lockdowns and closures on children’s physical activity and active play behaviours are evident. This study advances our knowledge of pandemic-associated impacts by sharing Ontario children’s and parents’ voices with regard to extended stay-at-home orders and their influence on movement-related behaviours and overall wellbeing (and how they differ for parents and children). In addition, the current study sheds light on the disruptive changes to family routines as a result of the pandemic, and how these have had significant impacts on children’s movement opportunities, and levels of social connectedness. This study identified barriers experienced, and facilitators used in regard to children’s physical activity during COVID times, as well as the influence of the pandemic on physical activity levels and perspectives on eventual return to sport/play. Further research is needed to unpack what type of supports can be put in place to ensure children receive enough activity if physical distancing and health measures remain in place, or in the case of additional waves of this, or another, pandemic. In addition, greater efforts are needed to ensure children’s voices are heard regarding the pandemic’s impacts on their activity levels and social connectedness.

## Supplementary Information

**Additional file 1: Appendix A.** Semi-Structured Interview Guide for Parent Interviews. **Appendix B.** Semi-Structured Interview Guide for Child Interviews.

## Data Availability

The data generated and analysed during the current study is available from the corresponding author on reasonable request.

## Abbreviations

WHO: World Health Organisation
MVPA: Moderate-to-vigorous physical activity
REB: Research ethics board

## References

[1] Felfe C, Lechner M, Steinmayr A (2016). Sports and Child Development. PLoS One.

[2] Carson V, Lee E-Y, Hewitt L, Jennings C, Hunter S, Kuzik N (2017). Systematic review of the relationships between physical activity and health indicators in the early years (0-4 years). BMC Public Health.

[3] Poitras VJ, Gray CE, Borghese MM, Carson V, Chaput J-P, Janssen I (2016). Systematic review of the relationships between objectively measured physical activity and health indicators in school-aged children and youth. Appl Physiol Nutr Metab.

[4] Tremblay MS, Carson V, Chaput J-P, Connor Gorber S, Dinh T, Duggan M (2016). Canadian 24-hour movement guidelines for children and youth: an integration of physical activity, sedentary behaviour, and sleep. Appl Physiol Nutr Metab.

[5] Carlsson E, Ludvigsson J, Huus K, Faresjo M (2016). High physical activity in young children suggests positive effects by altering autoantigen-induced immune activity. Scand J Med Sci.

[6] World Health Organization (2021). Coronavirus (COVID-19) events as they happen.

[7] Government of Canada (2021). List of Acts and Regulations - Canada.ca.

[8] Government of Ontario (2021). COVID-19 (coronavirus) in Ontario.

[9] de Lannoy L, Rhodes RE, Moore SA, Faulkner G, Tremblay MS (2020). Regional differences in access to the outdoors and outdoor play of Canadian children and youth during the COVID-19 outbreak. Can J Public Heal.

[10] Chaput J-P, Colley RC, Aubert S, Carson V, Janssen I, Roberts KC (2017). Proportion of preschool-aged children meeting the Canadian 24-Hour Movement Guidelines and associations with adiposity: results from the Canadian Health Measures Survey. BMC Public Health.

[11] Rhodes RE, Spence JC, Berry T, Faulkner G, Latimer-Cheung AE, O’Reilly N (2019). Parental support of the Canadian 24-hour movement guidelines for children and youth: prevalence and correlates. BMC Public Health.

[12] Moore SA, Faulkner G, Rhodes RE, Brussoni M, Chulak-Bozzer T, Ferguson LJ (2020). Impact of the COVID-19 virus outbreak on movement and play behaviours of Canadian children and youth: a national survey. Int J Behav Nutr Phys Act.

[13] Bates LC, Zieff G, Stanford K, Moore JB, Kerr ZY, Hanson ED (2020). COVID-19 Impact on Behaviors across the 24-Hour Day in Children and Adolescents: Physical Activity, Sedentary Behavior, and Sleep. Child (Basel, Switzerland).

[14] Pietrobelli A, Pecoraro L, Ferruzzi A, Heo M, Faith M, Zoller T (2020). Effects of COVID-19 Lockdown on Lifestyle Behaviors in Children with Obesity Living in Verona, Italy: A Longitudinal Study. Obesity (Silver Spring).

[15] Guan H, Okely AD, Aguilar-Farias N, Del Pozo CB, Draper CE, El Hamdouchi A (2020). Promoting healthy movement behaviours among children during the COVID-19 pandemic. Lancet Child Adolesc Heal.

[16] Xiang M, Zhang Z, Kuwahara K (2020). Impact of COVID-19 pandemic on children and adolescents’ lifestyle behavior larger than expected. Prog Cardiovasc Dis.

[17] Dunton GF, Do B, Wang SD (2020). Early effects of the COVID-19 pandemic on physical activity and sedentary behavior in children living in the U.S. BMC Public Health.

[18] Riazi NA, Wunderlich K, Gierc M, Brussoni M, Moore SA, Tremblay MS, et al. “You Can’t Go to the Park, You Can’t Go Here, You Can’t Go There”: Exploring Parental Experiences of COVID-19 and Its Impact on Their Children’s Movement Behaviours. 2021. 10.3390/children8030219. 10.3390/children8030219 PMC8000735 33809221

[19] Guba EG, Lincoln YS (1989). Fourth generation evaluation.

[20] Doyle S (2007). Member checking with older women: A framework for negotiating meaning. Health Care Women Int.

[21] Korstjens I, Moser A (2018). Series: Practical guidance to qualitative research. Part 4: Trustworthiness and publishing. Eur J Gen Pract.

[22] Anderson C. Presenting and evaluating qualitative research. Am J Pharm Educ. 2010;74(8):141. 10.5688/aj7408141. 10.5688/aj7408141 PMC2987281 21179252

[23] Braun V, Clarke V (2006). Using thematic analysis in psychology. Qual Res Psychol.

[24] Schumm WR (2021). Confirmation bias and methodology in social science: an editorial. Marriage Fam Rev.

[25] Mitra R, Cantello ID, Buliung RN, Faulkner GEJ (2017). Children’s activity-transportation lifestyles, physical activity levels and social-ecological correlates in Toronto, Canada. J Transp Health.

[26] Zenic N, Taiar R, Gilic B, Blazevic M, Maric D, Pojskic H, et al. Levels and Changes of Physical Activity in Adolescents during the COVID-19 Pandemic: Contextualizing Urban vs. Rural Living Environment. Appl Sci 202010. Available from: https://www.mdpi.com/2076-3417/10/11/3997

[27] Mitra R, Moore SA, Gillespie M, et al. Healthy movement behaviours in children and youth during the COVID-19 pandemic: Exploring the role of the neighbourhood environment. Health Place. 2020;65:102418. 10.1016/j.healthplace.2020.102418. 10.1016/j.healthplace.2020.102418 PMC7455528 32871499

[28] Nigg C, Oriwol D, Wunsch K, Burchartz A, Kolb S, Worth A (2021). Population density predicts youth’s physical activity changes during Covid-19 – Results from the MoMo study. Health Place.

[29] Elliott S, Drummond MJ, Prichard I, Eime R, Drummond C, Mason R (2021). Understanding the impact of COVID-19 on youth sport in Australia and consequences for future participation and retention. BMC Public Health.

[30] Truelove S, Bruijns BA, Vanderloo LM, O’Brien KT, Johnson AM, Tucker P (2018). Physical activity and sedentary time during childcare outdoor play sessions: A systematic review and meta-analysis. Prev Med (Baltim).

[31] Tremblay MS, Gray C, Babcock S, et al. Position Statement on Active Outdoor Play. Int J Environ Res Public Health. 2015;12(6):6475-6505. 10.3390/ijerph120606475. 10.3390/ijerph120606475 PMC4483712 26062040

[32] Schmidt SCE, Anedda B, Burchartz A, Eichsteller A, Kolb S, Nigg C (2020). Physical activity and screen time of children and adolescents before and during the COVID-19 lockdown in Germany: a natural experiment. Sci Rep.

[33] Salvy SJ, Roemmich JN, Bowker JC, Romero ND, Stadler PJ, Epstein LH (2009). Effect of peers and friends on youth physical activity and motivation to be physically active. J Pediatr Psychol.

[34] Barkley JE, Salvy S-J, Sanders GJ, Dey S, Von Carlowitz K-P, Williamson ML (2014). Peer influence and physical activity behavior in young children: An experimental study. J Phys Act Health.

[35] Beets MW, Vogel R, Forlaw L, Pitetti KH, Cardinal BJ (2006). Social support and youth physical activity: The role of provider and type. Am J Health Behav.

[36] Kracht CL, Sisson SB. Sibling influence on children’s objectively measured physical activity: a meta-analysis and systematic review. BMJ Open Sport Exerc Med. 2018;4(1) Available from: https://www.lib.uwo.ca/cgi-bin/ezpauthn.cgi?url=http://search.proquest.com/docview/2081944645?accountid=15115. 10.1136/bmjsem-2018-000405 PMC6196974 30364499

[37] Pelletier CA, Cornish K, Sanders C. Children’s Independent Mobility and Physical Activity during the COVID-19 Pandemic: A Qualitative Study with Families. International Journal of Environmental Research and Public Health. 2021;18(9):4481. 10.3390/ijerph18094481. 10.3390/ijerph18094481 PMC8122942 33922530

[38] Allender S, Cowburn G, Foster C (2006). Understanding participation in sport and physical activity among children and adults: a review of qualitative studies. Health Educ Res.

[39] Singh S, Roy D, Sinha K, Parveen S, Sharma G, Joshi G (2020). Impact of COVID-19 and lockdown on mental health of children and adolescents: A narrative review with recommendations. Psychiatry Res.

[40] Marques de Miranda D, da Silva AB, Sena Oliveira AC, Simoes-E-Silva AC (2020). How is COVID-19 pandemic impacting mental health of children and adolescents?. Int J Disaster Risk Reduct IJDRR.

