# Supplementary material for: Children and parents’ perspectives of the impact of the COVID-19 pandemic on Ontario children’s physical activity, play, and sport behaviours
Source: BMC Public Health. 2021 Dec 13;21:2271. doi: 10.1186/s12889-021-12344-w (PMC8666344; doi:10.1186/s12889-021-12344-w)
Supplement: Supplementary file 1 — Additional file 1: Appendix A. Semi-Structured Interview Guide for Parent Interviews. Appendix B. Semi-Structured Interview Guide for Child Interviews. [file 12889_2021_12344_MOESM1_ESM.docx]

**Appendix A – Semi-Structured Interview Guide for Parent Interviews**


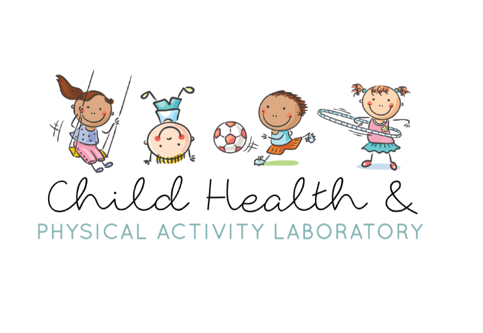

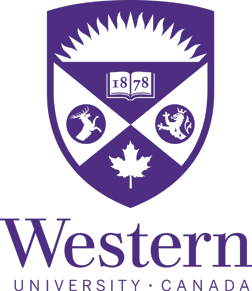


**Parents' Perspectives of their Child(ren)'s "Return to Play" Post-COVID-19 Pandemic**

**Semi-Structured Interview Guide for Parents**

Thank you for volunteering to participate in this online interview. We are here today to discuss your thoughts on your child(ren)’s return to active play opportunities post-COVID-19 pandemic. Specifically, we are looking to gather your intentions and opinions (i.e., fear, comfort) in regard to returning your child to both unstructured (i.e., playing in the neighbourhood) and structured (i.e., organized sport) play activities. Your feedback on this topic is very important.

The information collected today will complement the data our research team has obtained through online surveys from parents/guardians in Ontario. More specifically, your comments and in-depth responses will be used by our research team to further understand how increased supports can be put in place for young children and their parents/guardian’s post-COVID-19 pandemic.

There are no right or wrong answers. Everything discussed here today will be kept confidential, and all names will be removed from the transcripts and publications. By answering “yes” to the first question of the interview, you are providing consent to participate.

Are there any questions before we start?

1. Do you consent to participate in this interview?
2. What is your age and gender?
3. How many children aged 12 years and under do you provide care for?
   1. What are the ages of these children?
4. What types of active play/sports **outside of the home was** your child(ren) enrolled in **prior to COVID-19?**
   1. Did you and/or your child(ren) have a hard time adjusting to the new physical distancing measures that resulted in a cancellation of these activities?
   2. Did any of these activities continue in a virtual format during closures?
   3. Have your child and/or children already returned to active play/sport outside of the home (e.g., sports with other children)?
5. As a result of closures, what has been your **experience** with getting your child (or children) active during the COVID-19 pandemic?
   1. How ‘feasible’ (i.e., convenient and easy) was this for you while spending a large amount of time at home?
   2. In what way does your location influence your child’s return to play? (e.g., rural or urban)
   3. How receptive were your children to engaging in activity at home?
6. What **challenges** did you experience with getting your children active while at home?
   1. Please expand.
   2. In what ways did this impact you and your child(ren)?
   3. Do you feel that your children experienced challenges with the transition to more often home-based play and/or sport?
7. What **solutions** did you undertake to deal with these challenges?
   1. Please expand.
   2. Tell me more about that.
   3. How much time and effort did these solutions require?
8. If you haven’t already, **do you intend to make any changes to your child(ren)’s active play/sports programming as a result of the pandemic?**
   1. What sorts of changes? (e.g., withdrawal from team sport)
   2. What caused you to consider making these changes?
9. What are **your overall “feelings”** regarding your child(ren)’s eventual return to play/sports?
   1. Do you feel that the age of your child has an influence on these feelings?
      1. Do you think you would feel differently if your child was older/younger?
   2. What is making you feel concerned OR comfortable with regard to your child(ren)’s re-integration into play?
      1. Please expand.
   3. Are there any health precautions you would like to see in place that would make you feel more comfortable returning your child (if not yet returned)?
   4. Have you spoken with your child about their feelings in regard to returning to play?
      1. If yes, would you mind explaining how your child is feeling about returning to either active play or organized sport, or how they feel if already returned?

Thank you for participating!

**
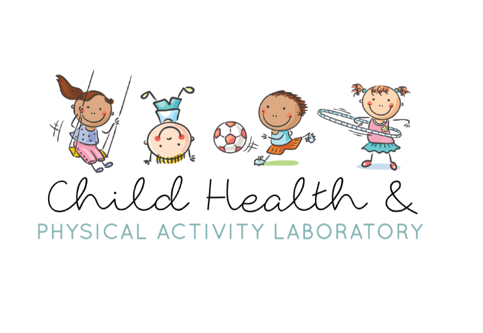
Appendix B - Semi-Structured Interview Guide for Child Interviews**

**
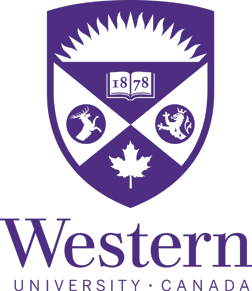
**

**Parents' Perspectives of their Child(ren)'s "Return to Play" Post-COVID-19 Pandemic**

**Semi-Structured Interview Guide for Children**

Thank you for volunteering to participate in this online interview. We are here today to talk about your thoughts on returning to active play and sports when the COVID-19 pandemic is over. Specifically, we are hoping you will tell us what you think about returning to both unstructured (e.g., playing in the neighbourhood) and structured (e.g., organized sports like hockey, soccer or dance) play activities. Your feedback on this topic is very important.

What we discuss today will allow our research team to better understand how COVID-19 has impacted your physical activity, and how you feel about returning to sport and play.

There are no right or wrong answers and everything you say today will be kept confidential. We will not use any names when we report out findings. By answering “yes” to the first question of the interview, you are agreeing to participate.

Are there any questions before we start?

1. Do you consent to participate in this interview?
2. How old are you?
3. What types of extracurricular activities/sports **outside of the home were** you enrolled in **prior to COVID-19?** (e.g., soccer, sports camps)
   1. Did you find yourself often thinking about or “missing” these activities?
      1. What did you miss about them?
4. Compared to your routine before the pandemic started (as a reminder, this was in March), do you think you were more or less active back then than you are now?
   1. Do you feel you got enough activity when you were spending a lot of time at home when the pandemic first started?
   2. Do you find it challenging to get active while at home?
5. What **challenges** did you experience with getting active while at home?
   1. Please expand.
   2. In what ways did these challenges impact you?
   3. If you experienced challenges, do you think it was hard for your parents, too?
6. What **helped you to** deal with these challenges?
   1. Please tell me more.
   2. Can you give me some examples of the types of activities you did often during the provincial lockdown? (for example, what did you do to get active when you were not allowed to go to school or sports practices?)
   3. Tell me more about that.
7. Have you and/or your parents/guardians already **made any changes as a result of COVID-19** to your active play or sport programming? For example, are you back in any team activities or planning to go back?
   1. What are some examples of these?
   2. What caused you to make these changes?
   3. Did you get to help your parents decide your plan for returning (or not) to sport/play?
   4. Tell me more about that.
8. **If you have** returned to sport/active play, what has it been like?
   1. Are there any changes to the activity (e.g., wearing masks) that you had to adjust to?
   2. Do you think these changes will be good or bad for your activity levels?
9. **If you haven’t returned**, how do you feel about your eventual return to play/sports programming?
   1. What excites you the most about returning?
   2. Does anything about returning make you feel nervous or uncomfortable? (e.g., new health protocols)

Thank you for participating!
